# Numerical Simulations to Predict the Seismic Performance of a 2-Story Steel Moment-Resisting Frame

**DOI:** 10.3390/ma13214831

**Published:** 2020-10-28

**Authors:** Roberto Tartaglia, Mario D’Aniello, Raffaele Landolfo

**Affiliations:** Department of Structures for the Engineering and the Architecture, University of Naples Federico II, 80100 Naples, Italy; mdaniel@unina.it (M.D.); landolfo@unina.it (R.L.)

**Keywords:** steel structures, moment resisting frame, beam-to-column joints, numerical analyses, non-linear analyses

## Abstract

The seismic response of steel moment resisting frames (MRFs) is influenced by the behavior of joints. Within the ongoing research project “FUTURE”(Full-scale experimental validation of steel moment frame with EU qualified joints and energy efficient claddings under Near fault seismic scenarios), shake table tests will be carried out on a two-story one bay MRF equipped with different types of prequalified beam-to-column joints. In order to design the experimental campaign, preliminary numerical simulations have been carried out to predict the seismic performance of the experimental mock-up in terms of distribution of damage, transient and residual interstory drifts. In this paper the main modeling assumptions and the results of the seismic analyses are shown and discussed. In particular, the response of joints was systematically investigated by refined finite element (FE) simulations and their behavior was taken into account in the global structural performance by means of both concentrated plastic hinge and distributed plasticity models. Both static and dynamic non-linear analyses show in which terms the type of models for plastic hinges influences the results. The modeling approach plays a key role only at very high seismic intensity where large ductility demand is imposed. In addition, changing the type of joints has less influence on the overall response of the frame.

## 1. Introduction

Steel moment resisting frames (MRFs) are commonly adopted in seismic areas. As deeply investigated in literature, the performance of MRFs is strictly related to the types of beam-to-column joints [1,2,3,4,5,6,7,8,9,10,11,12,13]. Indeed, MRFs equipped with partial strength beam-to-column joints can be characterized by excessive deformability and sensitivity to P-Delta effects as shown in [1,2,3,4,5]. On the other hand, the use of full strength and full rigid joints can lead to increase of constructional costs, especially when the column web panels have to be strengthened to prevent their plastic engagement [2,3,10,11]. The use of reduced beam sections (also commonly known as dog-bone joints) can significantly improve the seismic performance and reduce constructional costs [8,9,12]. In addition, dog-bone joints can be easily implemented to improve the performance of existing MRFs that are often designed without or with poor rules of hierarchy of resistances, as the case of ordinary frames in accordance with North American codes [13] or frames designed in ductility class low (DCL) in accordance with Eurocodes [2,10]. However, current Eurocodes [14,15] provide limited and noneffective rules to design seismic-resistant joints, because the hardening of plastic hinge as well as the increase of moment due to the shear force in the section where plastic hinge forms are not properly accounted for. In addition, current Eurocodes do not provide rules to guarantee the development of plastic deformations only in the ductile components of the joints as widely shown by [16,17,18,19,20].

Differently from Europe, in North America and Japan, the professional practice is oriented on the use of prequalified joints that are codified by specific standards [21] or recommendations [22].

In Europe, the practice of pre-qualified joints has been recently introduced following the results of recent research projects such as “EQUALJOINTS” and “EQUALJOINTS plus“ [23,24].

Besides the influence of joints, there has been substantial field evidence worldwide demonstrating that numerous local and global collapses of structural systems were caused by the devastating effects of the coupling between vertical and horizontal components of earthquake ground motions [25,26,27,28].

In addition, the design requirements of EC8 largely influence the response and the efficiency of steel MRFs. In particular, the drift limitations (i.e., damage limitation checks at serviceability limit state) often impose the increase of beam and column sizes to satisfy such requirement typically results in significant design over-strength and non-economical solutions [27]. This issue can be solved through the application of special ductile claddings that are able to accommodate large interstory drift ratios, higher than 1.5% [29,30,31]. The increase of lateral deformability may contribute to a greater sensitivity of the frames to P-Delta effects. However, it is well recognized that the current EC8 provisions concerning the control of P-Delta effects are very conservative and excessively penalizing for steel frames since it does not account for any source of structural overstrength due to randomness of the material resistance, the design overstrength, the hardening effects associated with redundancy, and the internal plastic redistribution [10,32].

The great overstrength of current EC8-compliant MRFs may also arise design difficulties to select commercial hot-rolled profiles for beams and columns [33] with the need to use welded built-up sections in the case of high-rise buildings or to optimize the structural system with ad-hoc solutions with floor truss beams made of tubular profiles [34]. Conversely, the large size of the structural member is beneficial for the durability against corrosion [35], thus leading to reduction of maintenance costs.

All issues abovementioned motivated the ongoing European research project “FUTURE” (acronym of full-scale experimental validation of steel moment frame with EU qualified joints and energy efficient claddings under near fault seismic scenarios). To overcome the discussed criticisms of the Eurocodes, shake table tests on a two-story mock-up will be carried out. The same mock-up is designed to be used in several experimental tests, after the substitution of damaged dissipative parts at the end of each test.

In this paper, the results of the preliminary numerical simulations to predict the seismic performances of the experimental mock-up are described and discussed.

The manuscript is mainly subdivided in three parts: In the first, the main design features of the mock-up are presented, then the results of the local FEM analyses and the calibration of the non-linear spring are described. Finally, the results in terms of both the static and dynamic analyses are illustrated.

## 2. The Investigated Structure

The investigated structure is a two-story frame with one bay in both the longitudinal and transversal direction with interstory height equal to 2.2 m. These geometrical features were sub-structured from a larger reference archetype building seismically designed in accordance with Eurocode 8. The mock-up was designed to resist both horizontal and vertical seismic action, allowing the easy replacement of all yielding and dissipative components, namely all moment resisting joints and the end portion of the base columns. The floor plan has a rectangular shape with a surface of 18 m^2^ (4.5 m in longitudinal and 4 m in transversal direction). MRFs are placed on the perimeter of the longitudinal direction, while in transversal direction, concentrically bracings are placed to provide torsional rigidity.

**Figure 1:**
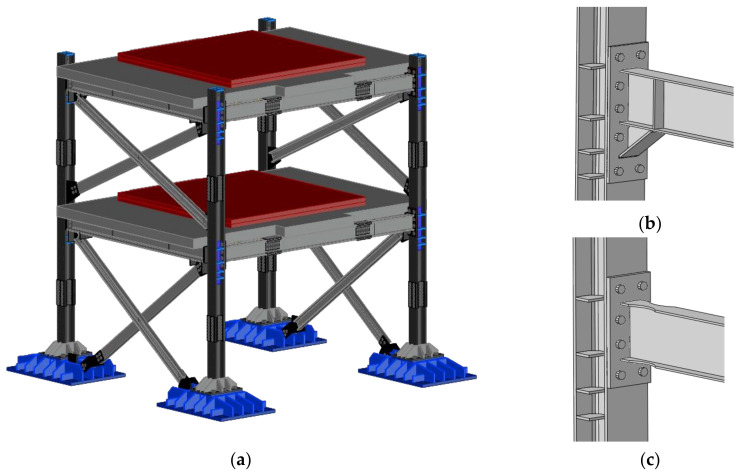
Experimental mock-up (**a**); the three investigated beam-to-column joints: haunched (**b**), reduced beam section (**c**).

The slab is made of a prefabricated solid reinforced concrete deck that is directly placed above the upper flange of the beams and connected to the steel flooring with pre-stressed threaded bars. Each steel flooring is made of two external longitudinal beams (IPE 240A), where the plastic hinge is expected, two internal longitudinal secondary beams (IPE 240A) which support the reinforced concrete slab, and two transverse external beams (HE260A) which support the slab and the internal longitudinal beams. HE 200B profiles are used for the columns. To replicate the dynamic behavior of the full-scale reference building, additional masses (in red in Figure 1) are tied on the concrete slab.

Detachable beam-to-column joints are designed and detailed as all-steel, thus preventing the composite action of the slab. In this preliminary phase, a full strength extended end-plate connection with reduced beam section (RBS), and another with full strength haunched (H) connection are considered. As deeply investigated in literature [23,24,25,26,27,28,29,30,31,32,33], these types of connection can provide an easy assembly and disassembly and an excellent ductility behavior.

The beam-to-column joints are designed to leave the column web panel free from plastic deformation. Therefore, supplementary web plates are directly welded on the column web panel. Torsional restraints were also introduced to avoid the lateral-torsional buckling of the beams. The cross sections of braces located in transverse direction are UPN 160. In addition, all static and dynamic analyses are performed considering two different modeling approaches to simulate the hysteretic behavior of the yielding zones, namely (i) concentrated plastic hinge (CPH) and (ii) distributed plasticity (DP). Therefore, starting from the same mock-up geometry, four different global models of the structure were analyzed (see Table 1).

## 3. Numerical Models

### 3.1. Modeling Assumptions

Two types of software were used to investigate the local behavior of the joints and then their influence on the global mock-up performance. First the beam-to-column and the column base joints were sub-structured from the mock-up and their response was individually simulated by means of Abaqus 6.14 [36]. Then, both distributed plasticity and concentrated plastic hinge (i.e., zero-length non-linear lumped spring) were calibrated in Seismostruct [37] against the results of finite element (FE) simulations.

**Figure 2:**
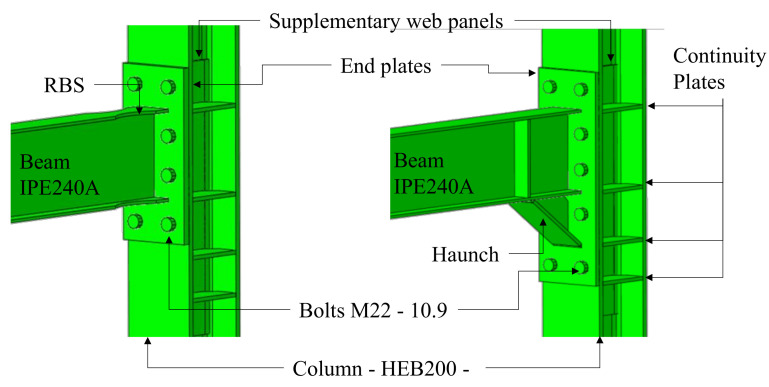
Geometrical features of the finite element (FE) models of the joints.

The main geometrical features of the FE models of the joints are shown in Figure 2. All FE modeling assumptions are discussed by authors in previous research [38,39]. However, for the sake of clarity, the main features of the FE models are reported hereinafter. All parts of the models were discretized using C3D8I elements (i.e., 8-node linear brick, incompatible mode) and the mesh dimension was chosen on the basis of preliminary sensitivity analyses.

The behavior of steel used for beams, columns, and plates was modeled as described in [38], where the average yield strength was set equal to 1.25*f*_y_ (being *f*_y_ the yield stress of the material) as defined by EN1998-1. The Von Misses yielding criterion was used together with the combined (i.e., both isotropic and kinematic) plastic hardening model.

The material properties of the bolts were modeled using a multilinear stress–strain curve as in [39]. In addition, the failure of the threaded area was simulated using the ductile damage model.

The geometrical imperfections of steel members were modeled though imposing the shape of buckling Eigen modes that is scaled up to the 80% of maximum allowable constructional tolerances.

The contacts between the surfaces were modeled by means of surface-to-surface interactions using both normal and tangential contact models.

**Figure 3:**
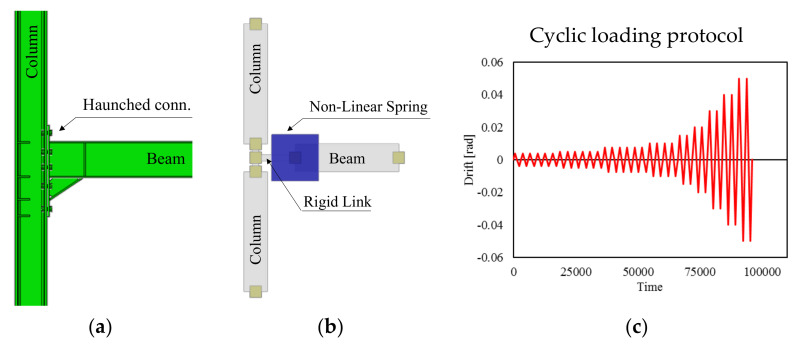
Abaqus (**a**) and Seismostruct (**b**) models to simulate the hysteretic response of the joints; adopted loading protocol (**c**) from AISC341-16.

Both monotonic and cyclic (the AISC41-16 [40] loading protocol, see Figure 3c, was adopted) analyses were performed on the investigated joints by imposing a displacement history at the tip of the beam segment for the beam-to-column joints and at the tip of the column segment for the column base joint. For this latter model, the presence of the vertical force was also accounted for.

The Seismostruct model is depicted in Figure 3b where the column panel zone dimensions were simulated using two rigid offsets in accordance with the scissors model (EN1993-8 [14]).

In the CPH model, the lumped non-linear spring was placed in the section where the plastic deformations are expected. The cyclic non-linear hysteretic response was mimicked by means of the smooth multilinear model available in the Seismostruct library. Contrariwise, a multilinear curve was introduced to replicate the joint monotonic response; in particular, in order to account for the cyclic degradation also in the monotonic analyses, the joint monotonic response was derived from the first cycle envelope curve of the simulated cyclic behavior.

The distributed plasticity (DP) model was developed by the introduction of a degrading stress–strain relation in the fibers of force-based (FB) distributed inelasticity elements. The advantage of this modeling approach is that it allows automatically taking into account the influence of the axial force on the column moment-rotation response. In addition, as observed by Chopra and McKenna [41], the distributed plasticity model is relatively insensitive to the type of damping model that could influence the results of dynamic analyses. Contrariwise, this approach does not allow to directly account for the cyclic degradation of strength and stiffness unless the equivalent material properties are calibrated a-priori [42].

**Figure 4:**
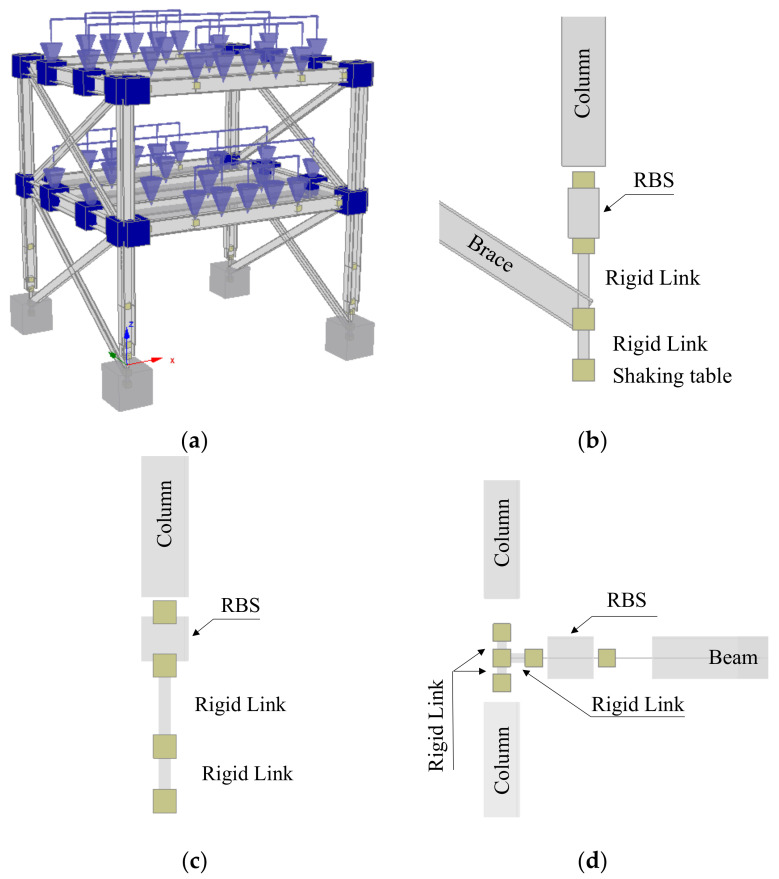
The global model (**a**), the detail of the column base in transverse direction (**b**), in longitudinal direction (**c**), and detail of the joint assembly with reduced beam section (RBS) (**d**).

The three-dimensional model of the structure was built in Seismostruct and it is shown in Figure 4. The diaphragm constraint was considered at each floor to simulate the in-plan rigidity of the slab. The masses were derived from the applied loads where also the contribution of the additional masses was accounted for. Figure 4b,c shows the details of the column base connection and the position of the reduced beam section (RBS) in transverse and longitudinal direction of the mock-up, respectively. It can be observed the presence of the two rigid links introduced to account for the real position of the brace system and its effect on the foundation. Figure 4d shows the details of the beam-to-column assembly with the reduced beam segment.

### 3.2. Simulation of the Hysteretic Response of the Joints

**Figure 5:**
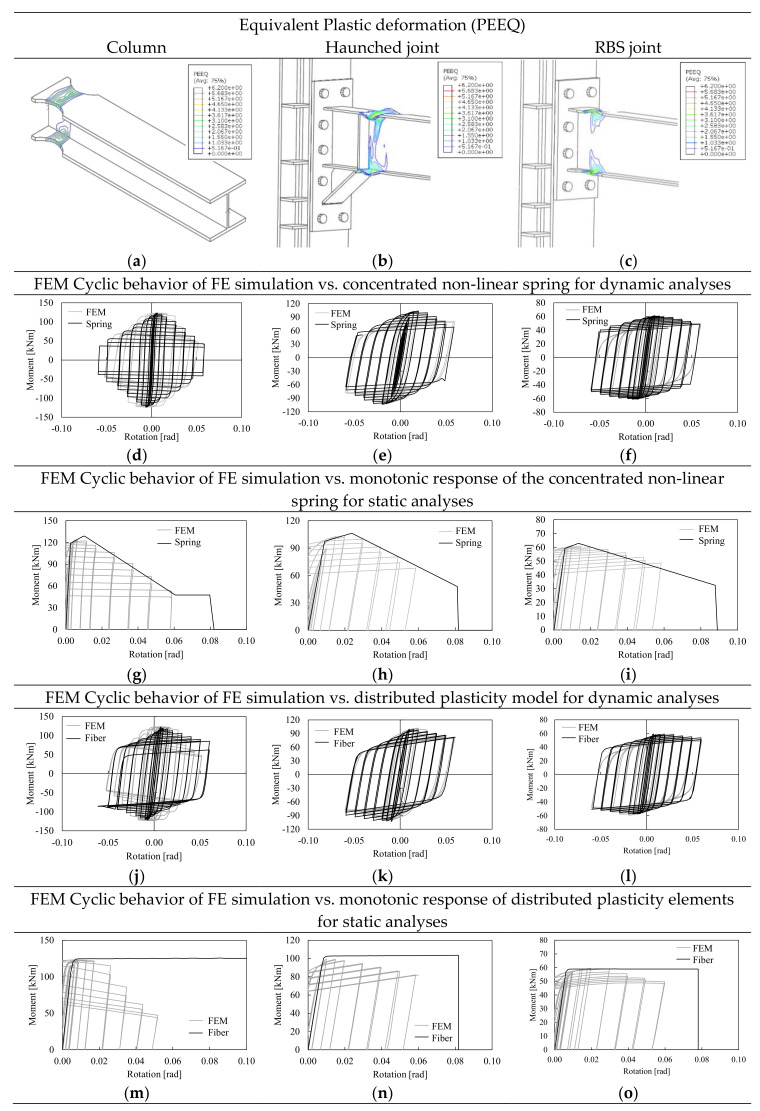
FE results in terms of equivalent plastic strain (PEEQ) (**a–c**) and comparison with concentrated non-linear spring (**d–i**) and distributed plasicity (**j–o**).

The FE results in terms of equivalent plastic deformations (PEEQ) and deformed shapes for all investigated joints are reported in Figure 5a–c. All joints show good cyclic behavior, thus confirming the design hypothesis. The model of the column base shows the concentration of all plastic deformations within the dog-bone segment. In addition, small shortening of the column (i.e., about 12 mm) was observed. This result is consistent with the experimental results presented by Lignos et al. [43], even if in the examined case, the small amount of vertical loads (i.e., about 0.1 times the axial resistance of column cross section) does not produce any squeezing of the segment where plastic deformations occur.

The haunched joint shows the concentration of all plastic deformations at the beam extremity, leaving in elastic range both the column web panel and the connection. In addition, due to the beam flange mill imperfections, it can be observed the activation of plastic buckling waves at large value of rotation. This phenomenon can be observed also from the moment rotation curve where, for large rotational value, a strength degradation can be observed. The RBS assembly preserves the connection and the column from plastic deformations that are concentrated within the dog-bone segment. In this case, since the beam flanges are cut, the plastic local buckling is triggered by the beam web and the strength degradation results smaller than what was observed for the haunched joints.

Figure 5d–o shows the comparison between the FE simulation vs. the models implemented for global analyses of the structure (i.e., CPH and DP). As it can be observed, both CPH and DP models are able to mimic the FE cyclic response curve in terms of elastic stiffness, yielding, and ultimate strength, as well as cyclic strength degradation (see Figure 5d–f for CPH and Figure 5j–l for DP). Figure 5g–i and Figure 5m–o show the comparison between the simulated first cycle envelope monotonic response and the cyclic response of the joints. As it can be easily recognized, the multilinear springs CPH are properly calibrated to reproduce also the joint cyclic degradation under monotonic loading. On the contrary, DP are not suitable to assess the seismic response of the structure using pushover analyses. This result depends on the type of modeling approach that is based on the integration of the stress–strain response at each fiber of the cross section, which relates the plastic behavior to the material constitutive law disregarding degradation phenomena as the local buckling of the parts in compression of the cross section. This limitation leads to the inaccurate simulation of the negative post-yield stiffness of the cyclic envelope, even if some recent studies show that this model can be profitably used if static cyclic non-linear analyses are performed [44,45]. Indeed, performing static cyclic analyses allows simulating the degradation that can be easily incorporated in the cyclic hardening law of the material model.

In the light of the obtained results, CPH are the most versatile type of modeling because they allow directly simulating the cyclic degradation for both static and cyclic non-linear analyses.

## 4. Numerical Prediction of the Seismic Performance of the Investigated Structure

The results of both static and dynamic non-linear analyses are hereinafter reported in terms of force-displacements response curves and distributions of interstory drift ratios for the cases with dog-bone and haunched joints.

### 4.1. Pushover Analyses

**Figure 6:**
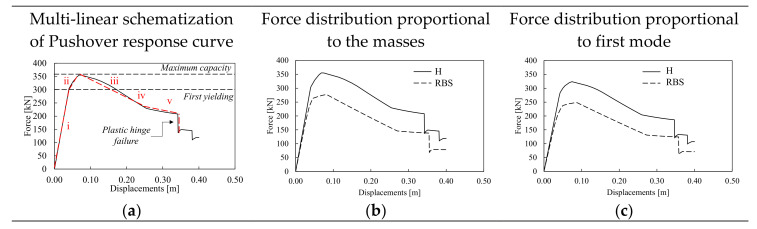
Pushover response curves of the structural model with the concentrated plastic hinge (CPH). Multi-linear schematization of Pushover response curve (**a**), Force distribution proportional to the masses (**b**), Force distribution proportional to first mode (**c**).

**Figure 7:**
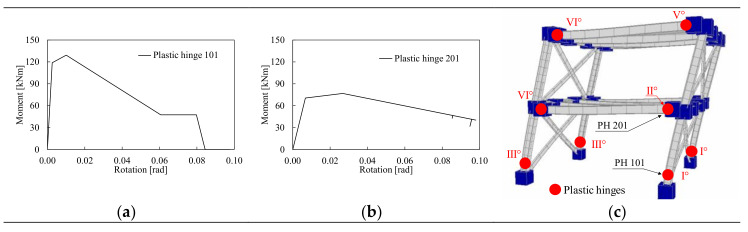
Pushover results of the structural model with haunched joint simulated with CPH in terms of column (**a**) and beam (**b**) moment rotation curves and distribution of plastic hinges (**c**).

**Figure 8:**
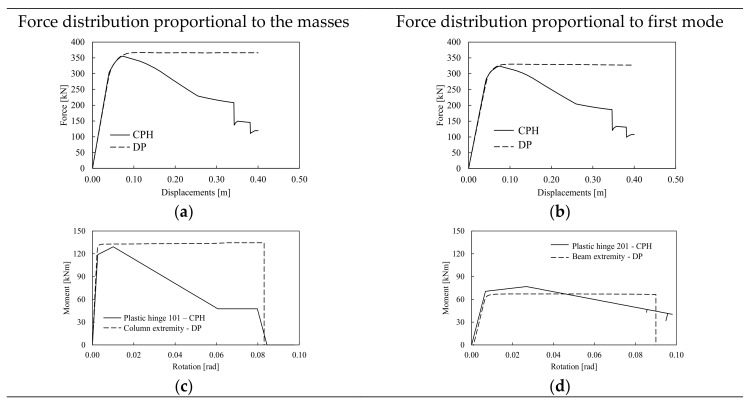
Pushover results in terms of force displacements (**a**,**b**) and moment rotation curves (**c**,**d**) of the structures modeled with distributed plasticity (DP).

The static non-linear performances of the investigated structures are shown in Figure 6, Figure 7 and Figure 8 in terms of force-displacements and moment-rotation curves. As recommended by the EN1998-1-1 [15], the pushover analyses were performed considering two vertical distributions of lateral forces, namely one proportional to the masses and another proportional to the shape of the first mode of vibration. The lateral increasing forces were applied at each node of the MRF at both side of the mock-up, while the average displacements of the nodes at second storey were monitored and plotted in Figure 6, Figure 7 and Figure 8.

Figure 6 shows the static non-linear response of the models with H and RBS joints simulated with CPH under force distributions proportional to the masses and the first mode of vibration of the mock-up. It can be observed that despite the differences in terms of elastic stiffness and peak resistance, the response curves of structures with H and RBS have similar shape. This result is mainly due to the fact that the moment-rotation relations of the plastic hinges were derived on cyclic simulations using the same loading protocol that affects the amount of cumulative damage and, consequently, the onset of degradation, thus explaining why different structures (i.e., the one with haunched joints and the other with RBS joints) experience similar values of the displacements at the peak of their response curve. Due to their similarities, the pushover response curves can be similarly schematized in five branches (see red dashed line in Figure 6a): (i) the elastic phase where all structural elements are in elastic range; (ii) the first yielding, where due to the activation of the first plastic hinges, the stiffness of the response curve changes; (iii) the third branch is the transition up to the peak resistance; (iv) the post-peak softening, whose slope is mainly governed by the hinge degradation and the second order effects; finally increasing the displacements demand, there is a variation of the softening slope that becomes flatter (v); which corresponds to the attainment of the residual resistance up to the failure of some plastic hinges.

To understand the influence of the local joint behavior on the frame response, the local behavior of one column base (i.e., PH101) and one beam at first storey (i.e., PH201) belonging to the H-CPH structure is plotted in Figure 7. The first plastic deformation develops within the columns at the base level (I), then increasing the horizontal actions the plastic deformations develop also in all the other plastic hinges (see Figure 7c). Figure 7a,b show that the first local failure is due to the column 101 that reaches its rotation capacity, while the beam is still on the softening branch.

The same analyses were conducted using DP models. Figure 8 depicts the pushover results comparing the response of the DP model (both in terms of force displacements and local moment-rotation curves) with the results obtained by the CPH models. As it can be observed, both elastic stiffness and lateral resistance are similar to the H-CPH structure. However, the pushover curves do not present any softening up to the ultimate displacements. The analyses were interrupted when the first elements reached their relevant maximum strain deformation. As formerly mentioned, the reason of the inaccuracy of DP models to simulate the softening of pushover curves depends on the features of the elements whose response is obtained by numerical integration of the stress–strain curve of the material models that are based on the virgin monotonic behavior of the material that is not affected by any cumulated damage.

### 4.2. Incremental Dynamic Analyses

A set of natural accelerograms was used to assess the dynamic response of the structure as well as to design the experimental campaign. Five signals were selected from PEER (Pacific Earthquake Engineering Research centre) and ITACA (ITalian ACcelerometric Archive) database with the basic requirement to have the peak ground acceleration in one longitudinal direction almost similar to the corresponding value in vertical direction. This choice depends on the feature of the testing equipment that is not suitable to adopt vertical and horizontal accelerations with scatter larger than 10%. For each investigated signal, only the longitudinal component with PGA closer to the vertical PGA and the vertical component were considered.

The number of five records descends from the difficulties to find other records that comply with the limits of the testing equipment, that are also strong enough to avoid scaling up with excessively high scaling factor (e.g., larger than 5).

In Table 2, the five considered earthquakes and their main features (i.e., site, magnitude (MS) and both vertical and horizontal PGA) are reported.

**Figure 9:**
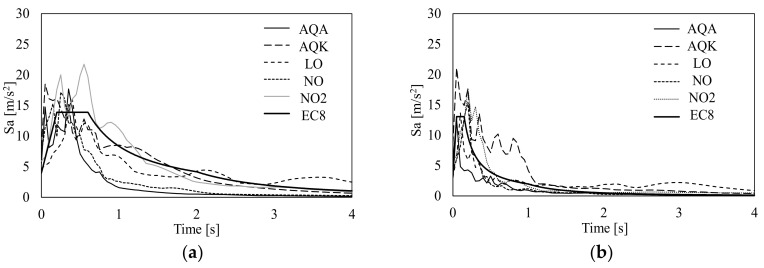
EC8 elastic spectrum vs. spectra of the selected records: horizontal (**a**) and vertical (**b**) components.

The incremental dynamic analyses (IDAs) were performed considering both horizontal and vertical components simultaneously; signals were properly scaled in the pseudo-acceleration value in order to have a good match between the pseudo-acceleration at the fundamental period of the mock-up given by Eurocode elastic horizontal spectrum and the corresponding ordinates of the spectra of the signals. The comparisons between the horizontal and vertical spectra are shown in Figure 9.

According to Eurocode 8, three limit state are defined namely damage limitation (DL), severe damage (SD), and near collapse (NC) and a different seismic intensity is associated to each of them. The ratio between the reference peak ground acceleration of the generic limit state as respect to the design level (i.e., SD limit state) is the factor that was considered to scale the seismic intensity in IDA in order to analyze the seismic response at each limit state. This factor varies as follows: 0.5 for DL, 1 for SD, and 1.73 for NC. Therefore, the IDAs were performed scaling the seismic intensity to cover also the cases corresponding to these three limit states. In addition, in order to assess the full performance of the mock-up, other intensities were considered. In detail, the IDAs were carried out scaling the signals as follows: 0.25, 0.50 (i.e., DL limit state), 0.75, 1 (i.e., SD limit state), 1.5, 1.73 (i.e, NC limit state), 2, 2.5, 3, 3.5, and 4 times the values of the acceleration of each record. The results are presented in terms of maxima (positive and negative) transient (IDR) and residual interstory drift (RIDR) ratios. Moreover, the structural dynamic behavior was plotted in terms of the maximum shear force with respect to maxima horizontal displacements.

**Figure 10:**
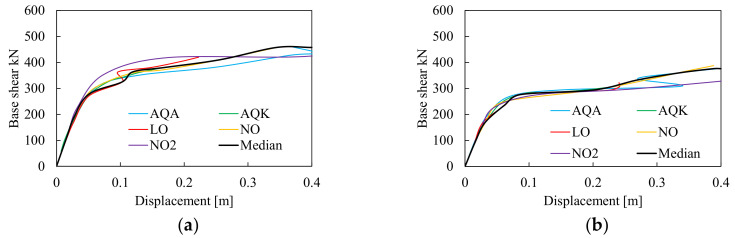
Incremental dynamic analyses (IDAs) curve of the frame equipped with haunched (H) (**a**) and RBS (**b**) joints modeled with CPH.

**Figure 11:**
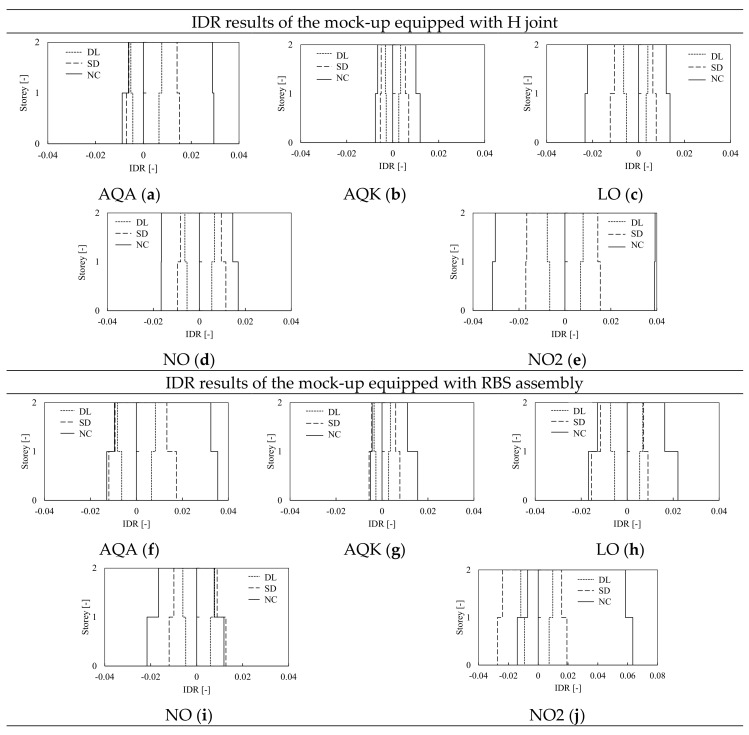
IDAs results in terms of inter-story drift ratio (IDR) of the frame equipped with H and RBS joints, modeled with CPH and subjected to AQA (**a,f**), AQK (**b,g**), LO (**c,h**), NO (**d,i**) and NO2 (**e,j**) accelerograms.

**Figure 12:**
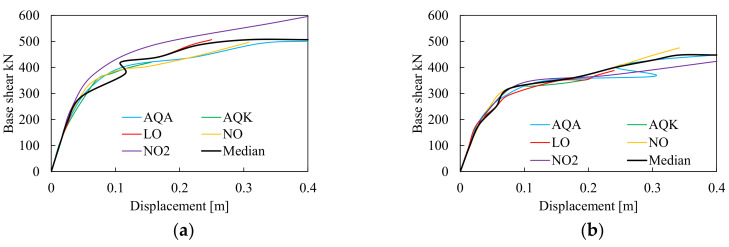
IDAs curve of the frame equipped with H (**a**) and RBS (**b**) joints modeled with DP.

**Figure 13:**
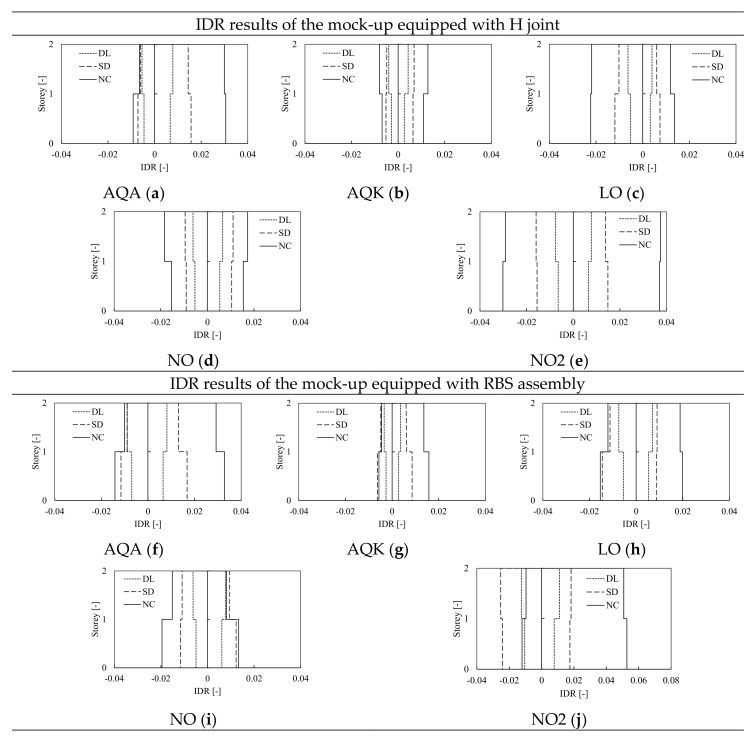
IDAs results in terms of interstory drift ratio (IDR) of the frame equipped with H and RBS joints, modeled with DP and subjected to AQA (**a,f**), AQK (**b,g**), LO (**c,h**), NO (**d,i**) and NO2 (**e,j**) accelerograms.

**Figure 14:**
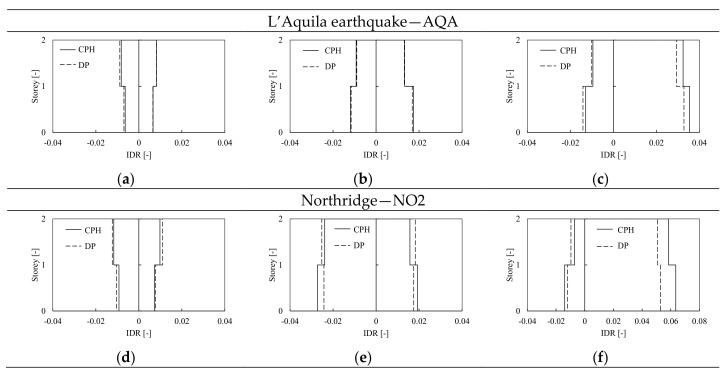
Comparison between the H frame modeled with CPH and DP at damage limitation (**a**,**d**), significant damage (**b**,**e**) and near collapse (**c**,**f**) limit state.

The IDAs were performed for all examined structures, also to investigate the influence of the modeling assumptions on the prediction of interstory drift ratios. The results of the frame equipped with H and RBS joints modeled with CPH are hereinafter depicted in Figure 10 and Figure 11, while the results of models with DP are depicted in Figure 12 and Figure 13. The comparison between the numerical predictions of CPH and DP are also shown in Figure 14.

In Figure 10 and Figure 12, the IDA curves for the models with CPH and DP are depicted in terms of maximum base shear resistance and corresponding horizontal displacements, where the black line is the median curve of all results. As expected, both H and RBS structures modeled with CPH show lateral resistance comparable with the pushover curves described in the previous paragraph. The results of the models with DP show greater lateral resistance mainly due to the amount of hardening simulated by the constitutive law of the material within the fibres of the cross section of DP elements. However, the analyses showed that in all cases, a global plastic mechanism occurs at a high level of the seismic intensity, which corresponds to top displacement greater than 0.1 m.

In Figure 11 and Figure 13, the IDR for each signal corresponding to the three scaling factors associated to the damage limitation (DL), significant damage (SD), and near collapse (NC) limit states are depicted. The results for the frame equipped with haunched joints modeled with CPH are summarized in Figure 11a–d, where it can be observed that the structure remains almost in elastic range in the case of DL and SD limit state. As confirmed by the small values of IDR (i.e., smaller than 0.02), small engagement of plastic zones is observed, being their response still in the pre-peak branch at NC limit state. However, large RIDR can be observed only if the mock-up is subjected to L’Aquila (AQA) and Northridge (NO2) signals, where the peak IDR at the first level are respectively equal to 3.2% and 4% of interstory height.

Similar considerations can be extended to the frame equipped with RBS joints and modeled with CPH. However, in this case, slightly greater IDR magnitude can be observed. This result is consistent with the design assumptions, being the frame with RBS weaker than the case with haunched joints. This aspect also explains the reason why the differences between the H and RBS models increase at higher seismic intensity (i.e., NC limit state), because the weaker frame experience larger damage and greater ductility demand to which is associated grater IDR demand.

Negligible residual drifts can be trivially observed when the structure does not show significant plastic deformations. Indeed, in the case of DL and SD limit state, the maxima evaluated residual drift ratios are smaller than 0.5% (see Table 3). Contrariwise, when the structure is subjected to large plastic demand, the corresponding residual drifts increase; thus, at near collapse (NC) limit state, larger value of residual drift can be observed. However, in the analyzed structures, only the analyses performed with AQA and NO2 signals (independently from the joint adopted H or RBS) show a residual drift close to 1%, while the structure can be considered as re-centered in all other cases.

The influence of the modeling approach was investigated comparing the results of the H-CPH structure to the same mock-up, but where the non-linear structural behavior was modeled through the distributed plasticity approach (DP). The models of frames with haunched and RBS joints equipped with DP elements show an overall response similar to the cases with CPH, even if the IDR demand is slightly smaller (as it can be observed comparing Figure 11, Figure 12 and Figure 13) due to the greater amount of hardening of the plastic components as well as the greater post-yield stiffness of DP elements. Indeed, as anticipated in Figure 5, differently from the pushover results, in the case of dynamic analyses, the joint strength degradation can be accounted for also by the DP model thanks to the cyclic material properties that should be properly calibrated. Figure 14 shows the comparison between the H-CPH and H-DP models when subjected to the AQA and NO2 signals at DL, SD, and NC limit states. The two differently modeled structures provide very similar results at both DL and SD limit states, independently from the type of signal. Indeed, in these cases, the structures behave almost in an elastic range, no appreciable differences can be pointed out. Contrariwise, at the NC limit state, some differences can be observed. In particular, the DP structures show slightly smaller IDR of the CPH model. Once again, this difference is mainly due to the beam plastic model. However, these differences are quite small and can be neglected at the global level within the range of DL to NC limit states.

## 5. Conclusions

Preliminary analyses to assess the seismic performance of a two-story steel experimental mock-up are presented. The main aims of the paper are the investigation of the accuracy and sensitivity of the modeling assumptions to simulate the seismic behavior of a steel frame with different types of moment resisting joints. To this end, the joint behavior was firstly simulated by means of refined FE model and then modeled in global structural model for the global seismic analyses by means of both concentrated plastic hinges and distributed plasticity elements. From the obtained results, the following conclusions can be drawn:

- FE simulations confirm that the non-linear performance of the examined joints is consistent with their relevant design assumptions.
- The adoption of concentrated plastic hinges (CPH) allows concentrating the plastic response of the joint in a lumped spring, while all other elements can be modeled as elastic elements. However, it should be noted that for the plastic hinge at the column base, the presence of vertical forces should be taken into account a-priori to properly calibrate the spring response.
- The smooth multilinear model was used to simulate the hysteretic behavior of CPH and it showed satisfactory agreement with the results obtained from FE simulations. Due to their versatility, this type of model can be easily used to mimic the joint non-linear behavior in both static and dynamic analyses.
- Distributed plasticity (DP) models allow to directly account for the bending-axial force interaction in the column. However, in case of static non-linear analyses, the DP model does not allow to account for the strength degradation. Contrariwise, both in cyclic static and dynamic analyses, the hinge strength degradation can be accounted for by the calibration of the cyclic degradation of the material model.
- The results from IDAs plotted in terms of maxima base shear and corresponding horizontal displacements are in line with the results of the pushover curves, showing almost the same lateral capacity and comparable elastic stiffness.
- Independently from the type of joints (i.e., H or RBS) and how they are modeled (i.e., CPH or DP), the investigated structures remain in elastic range up to the significant damage limit state. In addition, only when subjected to the Northridge earthquake, large plastic capacity (i.e., 4%) is required to the joints.
- The modeling approach plays a key role only at large value of imposed rotation, while no appreciable differences can be pointed out in elastic range, where CPH and DP provide almost the same results.
- The measured residual drift ratios are generally negligible (smaller than 0.5%) independently from the type of adopted joint (i.e., haunched or RBS); thus, only when the frame is subjected to severe plastic demand (i.e., in case of Northridge earthquake) residual drift ratios larger the 1% were measured.
- The study summarized in this paper represents the preliminary work to design the experimental campaign within the “FUTURE” project. Further research will be carried out to extend the numerical activity with parametric analyses to investigate the sensitivity of different modeling approaches on different geometrical and mechanical parameters such as the number of span, the number of storys, the interstory height, etc.

## Figures and Tables

**Table 1:** Investigated global models.

Label	Type of Beam-to-Column Joint	Modeling Approach
H-CPH	Haunched joint (H)	Concentrated plastic hinge (CPH)
H-DP	Haunched joint (H)	Distributed plastic model (DP)
RBS-CPH	Reduced beam section (RBS)	Concentrated plastic hinge (CPH)
RBS-DP	Reduced beam section (RBS)	Distributed plastic model (DP)

**Table 2:** The main features of the adopted accelerograms.

ID	Site	Date	MS	h	Station	Peak Ground Acceleration		
						Long.	Trans.	Vert.
-	-	-	-	km	-	g	g	g
AQA	L’Aquila, Italy	06/04/2009	6.3	8.8	AQA	0.44	0.40	0.47
AQK	L’Aquila, Italy	06/04/2009	6.3	8.8	AQK	0.35	0.33	0.37
LO	Loma Pietra, USA	18/10/1989	7.17	17	COR	0.47	0.64	0.43
NO	Northridge, USA	17/01/1994	6.7	12	ST24436	1.78	0.99	1.05
NO2	Northridge, USA	17/01/1994	6.7	12	NFS	0.59	0.58	0.55

**Table 3:** Evaluated residual interstory drift ratios (RIDR).

	Haunched Joints				RBS Joints		
	ID	DL	SD	NC	DL	SD	NC
	-	%	%	%	%	%	%
CPH	AQA	0.032	0.307	1.475	0.009	0.154	0.848
	AQK	0.015	0.032	0.013	0.009	0.001	0.151
	LO	0.01	0.135	0.581	0.003	0.087	0.524
	NO	0.013	0.067	0.262	0.041	0.080	0.367
	NO2	0.011	0.075	0.858	0.034	1.223	1.012
DP	AQA	0.031	0.316	1.424	0.008	0.149	0.806
	AQK	0.015	0.031	0.012	0.009	0.001	0.140
	LO	0.010	0.131	0.576	0.003	0.083	0.478
	NO	0.013	0.066	0.273	0.040	0.076	0.329
	NO2	0.011	0.074	0.836	0.033	1.174	0.921
